# Beyond Conventional: Italian Consumer Perceptions, Purchasing Habits, and Willingness to Pay for Ancient Grain Pasta

**DOI:** 10.3390/nu17142298

**Published:** 2025-07-11

**Authors:** Concetta Nazzaro, Anna Uliano, Marcello Stanco

**Affiliations:** Department of Law, Economics, Management and Quantitative Methods, University of Sannio, 82100 Benevento, Italy; cnazzaro@unisannio.it (C.N.); mstanco@unisannio.it (M.S.)

**Keywords:** ancient grains, Italy, consumer behavior, WTP, conjoint analysis

## Abstract

**Background/Objectives:** Ancient grains are increasingly recognized for their nutritional value, environmental sustainability, and connection to traditional agriculture. This study examines Italian consumers’ awareness, purchasing habits, and willingness to pay (WTP) for ancient grain pasta, focusing on the influence of product origin, price, and flour type on preferences. **Methods:** An online survey was conducted with 3020 Italian household grocery shoppers. Descriptive statistics assessed awareness and purchasing behavior, while conjoint analysis (CA) evaluated the relative importance of key product attributes (origin, price, and flour type) in pasta choices. The sample was segmented based on consumer knowledge of ancient grains. **Results:** A significant portion of respondents reported familiarity with ancient grains, perceiving them as “less refined” and “more digestible”; pasta emerged as the most purchased product. CA results indicated product origin as the most influential factor, followed by price, with flour type having comparatively lower influence. Notably, consumers more familiar with ancient grains showed a slight preference for ancient flour types and were less sensitive to price. **Conclusions:** While origin and price are primary drivers for pasta choices, knowledgeable consumers show greater valuation for flour type and accept higher prices. These findings provide strategic insights for stakeholders seeking to promote traditional, sustainable agri-food products through targeted marketing and transparent value communication.

## 1. Introduction

In recent years, consumer interest in agri-food products linked to territorial identity, sustainability, and health benefits has significantly increased, reflecting broader societal trends toward responsible consumption and cultural heritage appreciation [1,2,3,4]. Among these, products made from ancient grains have gained growing attention as a symbol of the rediscovery of traditional agricultural practices, environmental sustainability, and superior nutritional properties [5,6]. This shift aligns with the increasing demand for foods that benefit health and contribute to biodiversity conservation and rural area development [7,8,9]. In this regard, short and sustainable supply chains have been recognized as key drivers for enhancing resilience and supporting inner rural areas, particularly during times of systemic crisis [10].

Ancient grains, including varieties such as Kamut, Saragolla, and Senatore Cappelli, are characterized by their low environmental impact due to their adaptability to marginal lands and lower dependency on synthetic inputs, particularly in organic farming systems [9]. Their nutritional profile, which differs from that of modern wheat varieties, represents a key factor driving consumer interest, as these grains typically exhibit higher levels of micronutrients, antioxidants, and a distinct protein composition that may be associated with improved digestibility [11,12]. Furthermore, the resurgence of ancient grains aligns with the broader farm-to-table movement, prioritizing short supply chains and local sourcing to enhance food quality and authenticity [13,14].

Among ancient grain-based products, pasta has emerged as a particularly relevant product category. Pasta holds a unique place in Italian dietary culture, not only as a daily staple but also as an expression of national identity and culinary tradition [15,16], and also for its role within broader strategies of supply chain sustainability and value creation [17,18]. Italian consumers tend to be highly discerning regarding pasta, seeking qualities such as texture, taste, digestibility, and nutritional value [19,20,21]. In this context, the use of ancient grains may represent a compelling attribute capable of influencing consumer preferences. The nutritional and environmental benefits associated with ancient grains, combined with their historical and cultural connotations, may resonate with consumers’ pursuit of authenticity, health, and sustainability in food choices.

From a theoretical perspective, the growing consumer interest in ancient grain-based products intersects with several conceptual frameworks in the food and consumer behavior literature. The theory of perceived food quality is fundamental, positing that consumers evaluate food products not only on intrinsic attributes (e.g., taste, texture) but also on extrinsic cues such as origin, brand, and production methods. For traditional or niche foods, these extrinsic cues often act as symbolic indicators of quality, authenticity, and value, driving consumer evaluation and purchasing behavior [22,23]. In this context, ancient grains themselves serve as both an extrinsic and symbolic cue, potentially shaping consumers’ perceived quality and their willingness to pay (WTP) a premium. Previous research consistently shows that consumers are often willing to pay a premium for attributes conveying authenticity, sustainability, and health benefits in traditional or niche foods [24,25,26,27]. This is further supported by the literature on sustainable food consumption, which highlights the increasing importance of ethical and environmental motivations in shaping consumer choices [28,29,30,31]. These motivations often stem from a consumer’s underlying concern for the broader social and environmental impacts of their food choices.

Despite the growing consumer appeal, the market expansion of ancient grain-based pasta faces several challenges. First, the inherently lower yield of ancient grains compared to modern wheat varieties affects both production costs and final pricing [32,33]. Additionally, the limited availability of certified seeds and the absence of a structured supply chain contribute to market fragmentation, thereby reducing these products’ scalability and economic viability [9]. Another critical issue concerns food fraud and mislabeling, as the lack of standardized certifications has led to instances where products marketed as being made from ancient grains contain significant proportions of modern wheat, undermining consumer trust and distorting market dynamics [34,35].

From a consumer behavior perspective, several factors may influence purchasing decisions related to ancient grain pasta, including perceived health benefits, environmental considerations, taste preferences, and price sensitivity [36,37,38,39]. While these factors are well-documented drivers of food choices, a significant gap persists in understanding their relative importance, specifically within the context of ancient grain products. Even more critically, how these factors collectively influence consumers’ perceived value and their WTP for such niche products remains largely underexplored in the existing literature.

Furthermore, consumer choices for products like ancient grain pasta are also shaped by broader psychological and social constructs. For instance, health consciousness plays a crucial role; consumers who are highly aware of and prioritize health often seek out products with superior nutritional profiles, making ancient grains a compelling option [40,41]. Similarly, concerns for sustainability, environmental impact, and fair practices increasingly guide consumer behavior. The reduced environmental footprint and support for biodiversity associated with ancient grains directly resonate with these ethical considerations [42,43]. Furthermore, the concept of place identity can significantly influence preferences, as consumers often connect with products that embody regional heritage and traditional practices [44,45]. Ancient grains, deeply rooted in historical agricultural landscapes, can evoke a strong sense of place and authenticity, which may further drive consumer acceptance and premium valuation. Despite the recognized influence of these broader motivations on general food consumption, their specific interplay with consumer decisions regarding ancient grains, and their quantifiable impact on WTP, represents a critical unaddressed area.

Given this context and the identified gaps, this study aims to comprehensively investigate consumer attitudes towards products made with ancient grains, with a specific focus on pasta. It seeks to analyze consumers’ knowledge and purchasing habits regarding such products, particularly pasta, to better understand the impact of these multifaceted attributes on perceived pasta quality. Furthermore, the study aims to assess how these attributes, influenced by factors like health consciousness, ethical motives, and place identity, affect consumers’ purchasing decisions and their WTP.

Specifically, the following research questions are addressed:

RQ1. What are Italian consumers’ awareness, perceptions, and purchasing habits regarding ancient grains?

RQ2. To what extent does the ancient grain attribute influence Italian consumers’ pasta choices?

RQ3. What is consumers’ WTP for pasta made with ancient grains, and which factors influence it?

By answering these questions, this research aims to contribute to a deeper understanding of market dynamics and consumer decision-making in the context of sustainable and traditional agri-food products. The findings will be valuable for policymakers, producers, and marketers seeking to optimize product positioning, enhance transparency, and foster sustainable growth in the ancient grain sector.

## 2. Nutritional Profile and Health Aspects of Ancient Grains

Beyond their cultural and historical significance, ancient grains are increasingly recognized for their distinct nutritional profiles and potential health benefits, differentiating them from their modern counterparts. While modern wheat breeding has prioritized yield and processing efficiency, ancient varieties often retain a richer array of micronutrients, dietary fiber, and bioactive compounds, making them a valuable component of a healthy diet [9,11,46]. Specifically, ancient grains such as Kamut, Senatore Cappelli, and Timilia are characterized by higher concentrations of essential minerals like iron, zinc, magnesium, and selenium, which are crucial for various physiological functions [47,48]. They also typically boast a more diverse profile of antioxidants, including phenolic acids, flavonoids, and carotenoids, which contribute to their potent free-radical scavenging capabilities [48,49]. Furthermore, ancient grains often contain a higher proportion of dietary fiber, particularly soluble fiber, compared to modern refined wheat. This higher fiber content is associated with improved digestive health, slower glucose absorption, and a more favorable impact on gut microbiota diversity [50,51,52,53].

The potential health benefits associated with the consumption of ancient grains extend beyond their basic nutrient composition. Research suggests that the unique protein and starch structures found in ancient wheat varieties may contribute to improved digestibility for some individuals, particularly those with non-celiac wheat sensitivity [11,54]. While the term “lower gluten content” is often a misconception, studies indicate that the quality and structure of gluten proteins in ancient grains differ, potentially leading to varied immunological responses compared to modern wheat, although this remains an active area of research requiring further clinical investigation, especially for individuals with celiac disease [54,55]. Moreover, the higher antioxidant content in ancient grains has been linked to anti-inflammatory properties and a reduced risk of chronic diseases, including cardiovascular disease and type 2 diabetes, through mechanisms involving oxidative stress reduction and improved metabolic markers [11,55,56]. These findings underscore their potential role in public health nutrition strategies aimed at disease prevention.

The scientific evidence supporting the nutritional superiority and health-promoting potential of ancient grains aligns strongly with the evolving preferences of modern consumers [57,58,59,60,61]. As individuals increasingly seek foods offering health benefits, contributing to sustainable food systems, and possessing authentic origins, the attributes of ancient grains become particularly compelling [37,61,62]. Understanding such benefits is crucial for interpreting consumer perceptions and behaviors, and for informing effective public health and market strategies to promote the consumption of these nutritionally rich foods.

## 3. Materials and Methods

### 3.1. The Questionnaire

To achieve the study’s aims, an online questionnaire was administered using the Computer-Assisted Web Interviewing (CAWI) methodology to a representative sample of Italian household grocery shoppers. Such an approach includes several advantages, such as the lower costs compared to direct interviews, the possibility to include a large sample size, the speed and accuracy of data collection, and the absence of geographical limitations [63].

All participants acknowledged and agreed to an informed consent statement as a prerequisite for their involvement in the study. Prior to the main data collection, a pilot test involving 20 participants from the target population was conducted to identify ambiguities, resolve technical issues with the online platform, and gauge average completion time. The survey administration was entrusted to a market research company with extensive experience in consumer studies and market analysis. To ensure the sample’s representativeness, the company employed a rigorous quota sampling methodology. Quotas were carefully set based on the latest official demographic data for Italy, mirroring the national distribution across key variables such as gender, age groups, geographical macro-regions (North, Center, South/Islands), household size, and education level. Participants were recruited from their established consumer panel and screened to confirm their status as household grocery shoppers. The data collection process lasted two months, from June to August 2024, with an average response time of 10 min per participant. A brief message was showed at the start of the questionnaire, in order to mitigate the social desirability bias [64].

The design of the questionnaire was structured into multiple sections to capture various aspects of consumer behavior and preferences. The first section of the questionnaire aimed to collect information on consumers’ pasta purchasing habits, including questions on purchase frequency (e.g., ‘How often do you purchase pasta?’), and preferred pasta types (e.g., conventional, whole wheat, organic). The second section was focused on assessing consumer awareness and perceptions of ancient grains. Awareness was measured through a direct question (e.g., ‘Are you familiar with ancient grains?’) with predefined response options such as ‘Yes, I know them well,’ ‘I’ve heard of them but don’t know much,’ and ‘No, I don’t know them at all’. The third section of the questionnaire incorporated a conjoint analysis (CA) to assess consumer preferences regarding specific product attributes. According to Hair et al. [65], CA is a multivariate technique used to estimate or determine how respondents develop preferences. Such analysis is a widely used methodology in consumer research in agrifood studies, as it allows for the evaluation of how consumers trade off different product attributes when making purchasing decisions [66,67,68,69,70]. In this section, respondents were asked to choose between different pasta product profiles, each characterized by varying levels of the following attributes: origin, price, and the type of grain used in the pasta (flour type). The results of this analysis provided insights into the relative importance of each attribute in shaping consumer preferences and the trade-offs they are willing to make when purchasing pasta. The fourth section of the questionnaire focused on investigating consumers’ WTP for pasta made with ancient grains. This measurement tool offers a quantitative method for understanding the intricate factors that influence food preferences and views. The WTP estimates, in particular, reveal the price premium, which is the highest amount a current or potential consumer would pay for a specific product [64,71]. Respondents were presented with various pricing scenarios to evaluate their price sensitivity and assess the premium they would be willing to pay for pasta products featuring ancient grains. This section allowed for a better understanding of the demand for this type of product and provided insight into the potential market for pasta made from ancient grains.

The last section of the questionnaire gathered sociodemographic and psychographic data using validated scales to better understand the psychological and demographic profiles of the respondents. Specifically, the Ethical Food Choice Motives (EFCM) scale [72], the Healthy Lifestyle (HLS) scale [73], and the Place Identity (PI) scale [74] were included. For these multi-item scales, internal consistency reliability was assessed using Cronbach’s alpha. All scales demonstrated good reliability. These scales provided valuable insights into the values, lifestyle choices, and identity factors influencing consumer decisions in the context of food purchasing. The inclusion of these psychographic variables was crucial for testing the potential influence of ethical, health-related, and place-based considerations on consumers’ WTP for pasta made with ancient grains.

In addition, attention checks, known as trap questions, were included to ensure the quality of the responses [75]. A total of three trap questions were strategically embedded throughout the questionnaire to identify inattentive respondents. For instance, one question might instruct the respondent to select a specific, counter-intuitive option (e.g., ‘To show you are paying attention, please select “Strongly Disagree” for this statement’). These questions were designed to identify respondents who may not have been paying sufficient attention to the questionnaire. Respondents who answered any of the trap questions incorrectly were excluded from the data analysis, ensuring the reliability and accuracy of the final dataset.

### 3.2. The Conjoint Analysis Design

The CA design employed in this study aimed to evaluate consumer preferences for pasta made from ancient grains, focusing on three key attributes: origin, price, and flour type (Table 1). These attributes were selected based on their relevance to the emerging trends in consumer behavior regarding food quality and sustainability, as well as insights from previous studies in the field [6,76,77,78].

The first attribute, origin, was included to assess how consumers value the product’s geographical origin, particularly given the increasing interest in locally sourced and sustainable food products [77,79]. The levels considered for this attribute were Italy, European Union (EU), and extra-EU. These categories reflect the common classification of food origins in European markets, where “Made in Italy” is often perceived as a marker of quality and authenticity, while products from the EU may be associated with regulated standards, and non-EU products might carry concerns about sustainability or quality control. In this case, the ‘Italy’ level reflects not only a country of production but also a powerful cultural symbol associated with traditional methods, superior quality, and authenticity in food, as extensively documented in the study of Italian food culture [80].

Price is a crucial factor in consumer decision-making [81,82,83], and its inclusion allows for an understanding of how consumers balance price sensitivity with product quality. The three price levels chosen were low (EUR 1), medium (EUR 2.50), and high (EUR 4). These price levels were selected based on a preliminary market analysis to represent a typical range in the pasta market, ensuring the stimuli were both realistic and representative of current market conditions. Including a high price level allows for the exploration of premium product perceptions, which is particularly relevant in the context of ancient grains, a category often associated with higher production costs.

The flour type attribute was selected to capture consumer preferences related to the use of traditional versus modern grains in pasta production. The two levels for this attribute were conventional flour type and ancient grain flour type. This distinction is crucial as it aligns with growing consumer interest in health-conscious and sustainability-oriented food choices, particularly those focusing on ancient grains, which are perceived to offer nutritional and environmental benefits [9,13,84].

The experimental design initially produced 18 possible product profiles, which were further reduced to 9 final choice sets using an orthogonal array. This orthogonal design ensures that the attribute levels are independent, allowing for unbiased estimation of part-worth utilities—a measure of the relative importance of each attribute to respondents’ preferences [85]. The reduction in the number of choice sets from 18 to 9 was made to optimize the survey’s efficiency while maintaining statistical robustness. To avoid potential order bias, the nine choice cards presented to respondents were randomly ordered. A rating-based conjoint approach was employed, where respondents ranked each product profile on a scale from 1 to 9 (1 = ‘not preferable at all’ to 9 = ‘totally preferable’). This approach enables the estimation of individual preferences, which are then used to calculate the part-worth utilities and assess the relative importance of each attribute and the preference for different attribute levels.

### 3.3. Statistical Analysis

To assess the research aims, the data analysis was conducted in several steps. Initially, a descriptive analysis was performed to gain an understanding of the sample’s pasta purchasing habits, awareness of ancient grains, and the frequency of pasta consumption made from such grains. This allowed us to characterize the participants and identify general trends within the sample.

For assessing consumer preferences regarding specific attributes of pasta within the context of CA, part-worth utilities were estimated using Ordinary Least Squares (OLS) regression, a standard method in this field [86]. This approach enabled us to quantify the relative importance of each product attribute (origin, price, flour type) and determine how consumers value different levels of these attributes in pasta products.

To examine consumers’ WTP a premium for pasta made from ancient grains, data were stratified based on respondents’ familiarity with ancient grains. Specifically, respondents were classified into three groups: those who were familiar with ancient grains, those who had heard of them, and those who were not familiar at all. This categorization enabled us to assess whether awareness of ancient grains influences the premium that consumers are willing to pay. Subsequently, ANOVA and *t*-tests were performed to test for significant differences in the WTP across these three groups, allowing us to identify any notable variations in consumer preferences based on their level of awareness [87,88].

Finally, a linear regression model was applied to investigate the influence of sociodemographic and psychographic factors on WTP, with log-transformed WTP as the dependent variable. The use of a log transformation helped normalize the distribution of WTP, addressing issues of skewness and improving model fit [89]. The regression model included different independent variables, such as demographic and psychographic factors. OLS estimation was used to evaluate the effects of these variables on consumers’ WTP a premium for pasta made from ancient grains.

Data analysis was carried out using Stata 17, which allowed for effective handling of large datasets and provided the necessary tools for performing regression and statistical tests.

## 4. Results and Discussion

### 4.1. Descriptive Statistics

The sample includes 3020 individuals with an average age of 47.74 years (±14.52), ranging from 18 to 74 (Table 2). The gender distribution is balanced, with 48.77% identifying as male and 51.03% as female, while 0.20% identified as other. The average household size is 2.84 members (±1.17).

In terms of education, the majority of respondents hold a high school diploma (53.61%), followed by a university degree (29.34%), while 8.74% have a postgraduate qualification (master’s and/or PhD). Only 8.32% of the sample has completed primary or secondary education, indicating a relatively high level of education across the sample. Regarding employment status, nearly half of the respondents (49.01%) are employed, with an additional 12.12% working as freelancers. Students represent 7.05%, while housewives or households account for 9.04%. Pensioners comprise 14.67% and unemployed individuals represent 6.72%. Household income is concentrated in the low- to middle-income brackets, with 40.10% earning between EUR 16,000 and EUR 30,000, and 25.26% between EUR 31,000 and EUR 45,000. Notably, 19.27% of respondents reported an income below EUR 15,000, while only 15.37% earn above EUR 45,000 annually.

### 4.2. Focus on Ancient Grain Products

The consumption of ancient grain-based products has gained attention among Italian consumers, although their familiarity and purchasing habits still vary significantly. Approximately 33% of respondents report being well-acquainted with ancient grains, while 47% have only heard of them, and nearly 20% are unfamiliar. This suggests that, while awareness is growing, a significant share of respondents still lacks in-depth knowledge about these products. Among those who are familiar with ancient grains, Kamut stands out as the most recognized variety (77.48%), followed by Senatore Cappelli (52.12%) and Saragolla (11.08%) (Table 3).

Other varieties, such as Timilia and Romanella, are less widely known. This reflects the dominance of a few key ancient grain varieties in consumer awareness, with Kamut having a particularly strong presence in the market. Regarding the benefits related to the consumption of ancient grain-based products (Table 4), one of the most valued is that these products are less refined and more digestible, with a mean score of 5.67, indicating a strong consumer preference for the health benefits of these grains. Other important benefits include their potential to support small producers in fragile areas (5.53) and enhance local production (5.53), highlighting consumers’ appreciation for sustainability and local economic support. Additionally, the historical and cultural value of ancient grains (5.36) and their contribution to biodiversity (5.43) are also seen as significant, though slightly less so. These results suggest that, beyond health considerations, consumers value ancient grain products’ broader social and environmental impacts.

Regarding purchasing behavior, nearly 44% of respondents have previously purchased products made from ancient grains. Pasta emerges as the most commonly purchased item (chosen by 53.11% of the sample), followed by bread (49.96%) and flour (43.75%). In particular, the frequency of pasta consumption varies across the sample: while a substantial portion buys ancient grain pasta occasionally (70.10%), a smaller segment purchases it regularly (24.26%), and a few consumers buy it all the time (5.64%), indicating a growing interest but perhaps not yet a consistent habit.

Over 55% of respondents have never purchased products made from ancient grains. As shown in Table 5, several barriers continue to hinder the purchasing of such products. Lack of familiarity is cited as the most significant barrier, with 42.67% of respondents indicating that they did not purchase these products as they were unaware of them. Limited availability (35.13%) and high prices (30.27%) are also significant challenges, with many consumers noting that these products are not always easy to find or are too expensive.

Another barrier is that some consumers do not believe in the ethical claims associated with ancient grain production (5.34%), suggesting that perceptions around sustainability and local sourcing may not be universally trusted. Other factors, such as food intolerances and lack of attention to product characteristics, are less influential but still contribute to the reluctance to purchase ancient grain products.

These findings highlight the need for greater consumer education and improved accessibility, as many consumers express interest in these products but face challenges related to availability and price. Specifically, nutrition education initiatives should focus on communicating specific, verified health benefits (e.g., fiber content, potential digestibility) and ethical attributes (e.g., support for local producers, biodiversity). This education should be tailored to consumers’ varying levels of familiarity, leveraging their interest in health and sustainability. Furthermore, food labeling strategies could be optimized to clearly convey these nutritional and ethical attributes, empowering consumers to make informed choices and bridging the existing knowledge gap.

### 4.3. Conjoint Analysis Results

The CA results reveal insightful patterns regarding consumer preferences for pasta, specifically concerning the three key attributes of origin, price, and flour type. The mean part-worth utilities and mean relative importance values offer a comprehensive overview of how each attribute influences consumer choices.

The origin attribute is the most important factor in consumer decision-making, accounting for over 55% of relative importance (Table 6). This suggests that consumers place significant value on the geographical origin of the pasta. Among the levels, Italy holds the highest utility value (1.24), indicating a strong preference for Italian-origin pasta. This aligns with previous studies, showing that Italian products, particularly food, are often associated with higher quality, authenticity, and a sense of tradition [90,91,92,93]. The strong preference observed for pasta with ‘Italy’ as its origin is consistent with the well-established cultural significance of Italian food. This preference extends beyond simple geographical identification, reflecting consumers’ valuing of the authenticity, traditional heritage, and perceived quality intrinsically linked to the ‘Made in Italy’ label within the global food landscape [80]. Conversely, extra-EU origin carries a negative utility value (−1.21), highlighting a marked disfavor for products not originating from Italy or the EU. The EU origin level (−0.03) shows a neutral stance, suggesting that consumers may not strongly prefer EU products but are not overtly opposed to them either.

The price attribute is the second most influential factor in shaping consumer preferences, with a mean relative importance of 31.14%. The negative part-worth utilities for all price levels indicate that consumers are sensitive to price variations, with the lowest utility value observed for the EUR 4.00 price point (−1.72). This suggests that higher prices are a significant deterrent for consumers, even though premium products made from ancient grains may justify higher costs. The EUR 1.00 price level (−0.43) is the least negative, showing a moderate preference for more affordable products but still demonstrating a clear price sensitivity. The EUR 2.50 price point (−1.08) falls between the other two, representing a middle-ground preference. These results emphasize the importance of balancing product quality with price to appeal to a broader consumer base, especially in the context of pasta made from ancient grains, which may have higher production costs.

The flour type attribute, with a relative importance of 13.83%, is the least influential of the three attributes, but still relevant in shaping consumer preferences. The part-worth utilities for conventional flour type (0.03) and ancient grain flour type (−0.03) are nearly identical, indicating that consumers do not exhibit a strong preference for one over the other. This suggests that, at least in the context of this study, the use of ancient grains in pasta does not significantly sway consumer choices compared to conventional flour type. This finding might reflect a general lack of awareness or preference for ancient grains, which could be further explored in future research. It could also suggest that other factors, such as origin and price, might play a more dominant role in influencing consumer choices, overshadowing the specific choice of flour type.

### 4.4. Results Based on Consumer Knowledge of Ancient Grains

The results of the CA, segmented by the respondents’ knowledge of ancient grains, reveal interesting insights into how consumer preferences differ based on their familiarity with this product category. These findings highlight key differences from the overall results, allowing for a more nuanced understanding of consumer behavior. Below is a detailed comparison of the three sub-groups: those well-acquainted with ancient grains, those who only have heard about them and those unfamiliar with them (Table 7).

Respondents who are well-acquainted with ancient grains place the highest relative importance on the origin of the product (59.08%), with a clear preference for Italian products (mean utility of 1.31), and a strong disfavor for products from extra-EU regions (−1.28). In contrast, those with only basic knowledge of ancient grains show a slightly lower preference for origin (54.29%) and a reduced distinction between Italian and extra-EU origins. The least familiarity with ancient grains is associated with the lowest relative importance placed on origin (49.85%).

As for price, respondents with greater knowledge of ancient grains exhibit some price sensitivity, with a relative importance of 25.31% for price. However, they are less sensitive than other groups, as indicated by their moderate negative utility for higher prices, particularly for the EUR 4.00 price point. Conversely, consumers with only a basic awareness of ancient grains show a higher price sensitivity (33.08%), especially at higher price levels (−1.95 for the EUR 4.00 price). The most price-sensitive group is those unfamiliar with ancient grains (36.49%).

Regarding the preference for flour type, respondents well-acquainted with ancient grains show a slight preference for ancient grain flour type (mean utility of 0.04), whereas those with less familiarity exhibit a preference for conventional flour type. This is particularly true for those unfamiliar with ancient grains, who show a clear preference for conventional flour type over ancient grain flour type, with a negative utility for the latter (−0.12).

Overall, the results suggest that familiarity with ancient grains influences how much weight consumers place on product attributes such as origin, price, and flour type. Those with a better understanding of ancient grains are more likely to prioritize origin and are willing to pay a premium, while consumers unfamiliar with the product tend to be more sensitive to price and prefer conventional options. This highlights the need for targeted marketing strategies that emphasize the benefits of ancient grains for different consumer segments based on their level of familiarity.

### 4.5. Analysis of Consumers’ WTP for Ancient Grain Pasta

The analysis of consumers’ WTP a price premium for pasta made from ancient grains reveals that 2651 out of 3020 respondents (approximately 88%) declared a positive WTP, with an average premium of EUR 1.12 (±0.94). When disaggregating this figure by levels of product familiarity, clear differences emerge. Consumers who report being well-acquainted with ancient grains (*n* = 957) are willing to pay, on average, a significantly higher premium (EUR 1.37), compared to those who have only heard of them (EUR 1.02), and those who are entirely unfamiliar (EUR 0.87). These differences are statistically significant, as confirmed by an ANOVA test (F = 59.22, *p* < 0.001) and pairwise *t*-tests, rejecting the null hypothesis of equal means across knowledge levels.

This gradient in WTP aligns with findings from previous studies highlighting the influence of consumer awareness and perceived product benefits on premium pricing decisions [94,95]. Individuals more familiar with ancient grains are likely to associate them with health-related and ethical attributes, such as digestibility, biodiversity preservation, and support for local producers, which may enhance their perceived value and justify a higher price point. Conversely, unfamiliar consumers may lack the information necessary to make value-based judgments and thus exhibit greater price sensitivity. This result reinforces the relevance of information asymmetry in the adoption of niche, sustainable food products [96,97,98].

To further explore the drivers of this premium, a linear regression on the natural logarithm of WTP (ln_WTP) reveals that income, age, and HLS are the strongest predictors (Table 8). Higher income levels are associated with greater WTP, supporting prior research that links ethical food choices to economic resources [64,99,100]. A negative association with age suggests that younger consumers may be more responsive to values such as sustainability, innovation, or dietary novelty. This result is consistent with previous contributions, such as those of Kovacs and Keresztes [101], who highlight a greater openness among younger individuals toward environmentally conscious consumption, and Halicka et al. [102], who report a stronger preference for novel and health-oriented foods in younger demographic groups. The strongest effect is observed for HLS, confirming that consumers with a healthier lifestyle are significantly more inclined to support food choices aligned with wellness and naturalness [64,103], attributes commonly associated with ancient grains.

Interestingly, EFCM displays only a weak and marginally significant effect, hinting that while ethics may matter, they are secondary to more immediate personal health concerns. PI, which reflects attachment to local territory and culture, does not appear to significantly impact WTP, contrary to what might be expected given the cultural framing of ancient grains. Other sociodemographic variables, such as gender, education, and household size, are also not significant, suggesting that motivations for purchasing ancient grain products are more behaviorally and value-driven than demographically determined.

These findings highlight a differentiated market structure: informed, health-conscious, and financially capable consumers are more willing to pay a premium for ancient grain products, while others remain skeptical or price-sensitive. This segmentation underlines the importance of targeted marketing strategies that emphasize health, quality, and sustainability to appeal to high-WTP consumers, while possibly reducing price or increasing accessibility for less-engaged segments.

## 5. Limitations of the Study

Despite the robustness of the findings, this study has certain limitations that warrant consideration when interpreting the results.

First, the cross-sectional design of our study limits the ability to draw definitive causal inferences. While we identified associations and perceptions, we cannot establish direct cause-and-effect relationships between variables. This means, for instance, that while we observe a preference for certain ancient grain attributes, we cannot definitively say that exposure to information about these attributes causes a change in purchasing behavior over time.

Second, our reliance on self-reported measures introduces the potential for social desirability bias. Participants might have provided answers they perceived as more socially acceptable or desirable, rather than reflecting their true perceptions or behaviors, particularly concerning health-conscious choices or sustainable practices. More specifically, concerning WTP, our use of self-reported data elicited through direct questions carries inherent limitations, such as potential hypothetical bias. Stated intentions to pay may not perfectly align with actual purchasing behavior in a real market scenario. This could lead to an overestimation or underestimation of true WTP, as respondents might declare a higher WTP for perceived benefits than they would actually commit to when faced with a real purchasing decision.

Furthermore, while our sample was diverse, it may not fully capture the regional or cultural heterogeneity within Italy. Different regions may have unique culinary traditions, perceptions of food, or economic realities that could influence consumer preferences and WTP for ancient grain pasta. This limits the generalizability of our findings across the entire Italian population and suggests that consumer segments not adequately represented might exhibit different patterns of behavior or preferences.

Finally, while rating-based conjoint analysis is effective for estimating granular attribute preferences, it does not fully replicate actual purchasing decisions where consumers choose among competing product alternatives in a naturalistic setting. This methodological choice inherently limits its direct applicability for precise market share predictions. The absence of a competitive context means that the estimated utilities and WTP values reflect preferences in an isolated environment, and might not perfectly translate to real-world purchasing decisions where trade-offs against a broader range of products (including conventional options or other alternative flours) are made. This could lead to an inflated perception of the importance of certain attributes compared to how they would perform in a competitive market.

These limitations highlight areas for future research, including longitudinal studies, experimental designs to test actual purchasing behavior, and broader sampling strategies to enhance the external validity of findings.

## 6. Conclusions

This study aimed to explore Italian consumers’ attitudes, preferences, and WTP for products made from ancient grains, focusing on pasta, and to identify the key factors influencing their choices in this emerging market. Specifically, it reveals that while awareness of ancient grains is growing among Italian consumers, in-depth familiarity still varies significantly, with a notable portion remaining unaware or only vaguely familiar (RQ1). Moreover, a substantial majority of consumers (approximately 88%) are willing to pay a price premium for ancient grain pasta, with this willingness significantly increasing among those more familiar with these products (RQ2). Furthermore, the analysis of WTP drivers demonstrates that income, age (negatively associated), and a healthier lifestyle (HLS) are the most robust predictors of this premium, indicating a market segmentation driven more by behavioral and value-based factors than by general demographics (RQ3). Furthermore, the findings reveal that familiarity with ancient grains significantly shapes consumer behavior, particularly influencing perceived product value, price sensitivity, and preferences regarding origin and ingredient type. While health-related and socioenvironmental attributes are generally appreciated, their impact on purchasing behavior is mediated by consumers’ level of knowledge. Origin emerged as the most influential product attribute, underscoring the central role of territorial identity and quality perception in food choices. Conversely, while flour type has some relevance, it remains secondary, particularly among consumers less familiar with ancient grains.

From a theoretical standpoint, this study contributes to the existing literature on consumer food choice by highlighting the nuanced interplay between cognitive factors (e.g., consumer knowledge and familiarity), affective factors (e.g., perceived quality and trust in origin), and conative aspects (e.g., WTP and preference for specific attributes) within the context of niche food markets. The identified mediating role of consumer knowledge further underscores the importance of information processing in shaping preferences for attributes that often carry credence qualities. Furthermore, the strong influence of origin reinforces theories on consumer ethnocentrism even for seemingly ‘simple’ products like pasta, suggesting that cultural and geographical identity can be a potent driver in consumers’ decision-making processes.

From a managerial perspective, the results of this study offer key actionable insights for stakeholders in the ancient grain pasta market. Firstly, given that consumer familiarity significantly shapes preferences, investing in comprehensive consumer education and targeted communication strategies is paramount. These initiatives should not only build general awareness but also specifically emphasize the distinct nutritional and ethical benefits of ancient grains. For instance, nutrition education campaigns could highlight the unique dietary fiber content, potential lower glycemic index, or specific micronutrient profiles of ancient grains compared to modern varieties, grounded in scientific evidence. Furthermore, the pre-eminent role of ‘origin’, particularly ‘Italian’, underscores the strategic importance for producers and marketers to leverage geographical indications and national identity in branding and communication. Emphasizing the local sourcing and traditional heritage of Italian ancient grain pasta can significantly enhance perceived product value and drive purchasing decisions.

In line with the study’s results, the identified consumer preferences for healthier, identity-linked, and sustainable products suggest a strategic opportunity for developing short supply chains of ancient grains. This approach can foster more sustainable, equitable, and localized food systems, contributing significantly to agricultural biodiversity and rural regeneration. These efforts align with broader public health nutrition strategies aimed at promoting diversified and resilient food supplies, which are crucial for enhancing population health and food security, especially in the face of climate change and biodiversity loss. Therefore, supporting local supply chains, enhancing product visibility, and incentivizing sustainable agricultural practices could be crucial for policymakers to promote broader consumer adoption and realize the full economic and regenerative potential of these valuable crops. Ultimately, these insights suggest that organizing the ancient grain supply chain could benefit from an integrated policy approach that connects economic, regulatory, and territorial tools to valorize agricultural commons and generate shared local value, recognizing ancient grains as cultural and relational goods capable of promoting alternative production and consumption models.

## Figures and Tables

**Table 1:** Product’s attributes and attribute levels.

Attribute	Attribute Level
Origin	Italy
	EU
	Extra-EU
Price	Low (EUR 1.00)
	Medium (EUR 2.50)
	High (EUR 4.00)
Flour type	Conventional
	Ancient grains

Conventional semolina refers to durum wheat semolina commonly available on the market, typically from modern or widely cultivated wheat varieties, as opposed to ancient grains.

**Table 2:** Sociodemographic characteristics of the sample (N = 3020).

Variable	Mean	Freq. (%)	Std. Dev.	Min.	Max.
**Age**	47.74		14.52	18	74
18–25 y.o.		9.75			
26–35 y.o.		15.02			
36–45 y.o.		19.18			
46–55 y.o.		22.85			
56–65 y.o.		19.13			
Over 65 y.o.		14.07			
**Gender**					
Male		48.77			
Female		51.03			
Other		0.20			
**Household size**	2.84		1.17	1	6
1 member		12.38			
2 members		29.90			
3 members		26.95			
4 members		23.77			
Over 5 members		6.99			
**Education**					
Primary school		0.17			
Secondary school		8.15			
High school		53.61			
University degree		29.34			
Master’s and/or PhD		8.74			
**Job**					
Employee		49.01			
Freelancer		12.12			
Student		7.05			
Housewife/household		9.04			
Unemployed		6.72			
Pensioner		14.67			
Other		1.39			
**Family income**					
EUR < 15,000		19.27			
EUR 16,000–30,000		40.10			
EUR 31,000–45,000		25.26			
EUR 46,000–60,000		9.11			
EUR > 60,000		6.26			

**Table 3:** Familiarity with different ancient grain varieties among respondents.

Ancient Grain	Percent (%)
Kamut	77.48
Senatore Cappelli	52.12
Saragolla	11.08
Timilia	10.97
Romanella	10.65
Marzellina	7.48
Jervicella	4.07
Ianculedda	3.90
Other	3.78

**Table 4:** Benefits associated with ancient grain products, ordered by relevance.

Variable	Mean	Std. Dev.	Min.	Max.
They are less refined and therefore more digestible	5.67	1.38	1	7
They support small producers working in fragile areas (e.g., inner areas)	5.53	1.40	1	7
They contribute to enhancing local productions	5.53	1.41	1	7
They are a valuable source of biodiversity	5.43	1.43	1	7
They strengthen the historical and cultural value of the territories	5.36	1.50	1	7
They contain less gluten	4.73	1.72	1	7

**Table 5:** Barriers to the purchase of ancient grain-based products (N = 1685).

Variable	Percent (%)
Lack of familiarity	42.67
Limited availability	35.13
High costs	30.27
Lack of attention to product characteristics	9.38
Lack of trust in their ethical claims	5.34
Food intolerances	2.61

**Table 6:** The part-worths of attribute levels and relative importance of attributes.

Attribute	Levels	Mean Part-Worth Utility	Mean Relative Importance (%)
Origin			55.03
	Italy	1.24	
	EU	−0.03	
	Extra-EU	−1.21	
Price			31.14
	EUR 1.00	−0.43	
	EUR 2.50	−1.08	
	EUR 4.00	−1.72	
Flour type			13.83
	Conventional	0.03	
	Ancient grains	−0.03	

**Table 7:** Part-worth utilities and relative importance of product attributes by consumer knowledge of ancient grains.

Attribute	Level	Well-Acquainted with Ancient Grains (1010)		Has Only Heard of Ancient Grains (1423)		Unfamiliar with Ancient Grains (587)	
		Mean Part-Worth Utility	Mean Relative Importance (%)	Mean Part-Worth Utility	Mean Relative Importance (%)	Mean Part-Worth Utility	Mean Relative Importance (%)
Origin			59.08		54.29		49.85
	Italy	1.31 ***		1.22 ***		1.18 ***	
	EU	−0.03 *		−0.04 *		−0.01 *	
	Extra-EU	−1.28 ***		−1.18 ***		−1.17 ***	
Price			25.31		33.08		36.49
	EUR 1.00	−0.30 ***		−0.49 ***		−0.53 ***	
	EUR 2.50	−0.74 ***		−1.22 ***		−1.32 ***	
	EUR 4.00	−1.19 ***		−1.94 ***		−2.11 ***	
Flour type			15.62		12.63		13.66
	Conventional	−0.04 *		0.05 *		0.12 **	
	Ancient grains	0.04 *		−0.05 *		−0.12 **	

* *p* < 0.10; ** *p* < 0.05; *** *p* < 0.01.

**Table 8:** Results of the linear regression.

ln_WTP	Coef.	St. Err.	t-Value	*p*-Value	[95% Conf. Interval]
Household size	0.00	0.01	0.14	0.89	−0.03–0.03
Education	0.02	0.02	1.08	0.28	−0.02–0.06
Income	0.06	0.02	3.79	0.00 ***	0.03–0.09
Gender	0.03	0.03	0.92	0.36	−0.03–0.09
Age	−0.01	0.00	−4.50	0.00 ***	−0.01–−0.00
HLS	0.10	0.02	5.07	0.00 ***	0.06–0.13
PI	0.01	0.01	0.63	0.53	−0.02–0.03
EFCM	0.03	0.02	1.86	0.06 *	−0.00–0.06
Constant	−0.86	0.13	−6.53	0.00 ***	−1.11–−0.60

*** *p* < 0.01, * *p* < 0.1.

## Data Availability

Dataset available on request from the authors. Datasets are not publicly available due to time limitations.

## Abbreviations

The following abbreviations are used in this manuscript:

WTP	Willingness to Pay
CA	Conjoint Analysis
EFCM	Ethical Food Choice Motives
HLS	Healthy Lifestyle
PI	Place Identity
